# Low-Iron Bovine Lactoferrin Affects Adhesion, Erythrophagocytosis, Cytotoxicity, and Proteolytic Activity in *Entamoeba histolytica* Trophozoites

**DOI:** 10.3390/ijms27125257

**Published:** 2026-06-10

**Authors:** Magda Reyes-López, Christian Ávalos-Gómez, Gerardo Ramírez-Rico, Jesús Serrano-Luna, Mireya de la Garza

**Affiliations:** 1 Departamento de Biología Celular, Centro de Investigación y de Estudios Avanzados (Cinvestav-IPN), Ave. IPN 2508, Mexico City 07360, Mexico; magda.reyes@cinvestav.mx (M.R.-L.); chris8814@hotmail.com (C.Á.-G.); jesus.serrano@cinvestav.mx (J.S.-L.); 2 Laboratorio de Patología Molecular Veterinaria, Facultad de Estudios Superiores de Cuautitlán, Universidad Nacional Autónoma de México, Cuautitlán Izcalli 54714, Estado de Mexico, Mexico; garmvz@gmail.com

**Keywords:** *Entamoeba histolytica*, bovine lactoferrin, cytotoxicity, proteolytic activity, erythrophagocytosis, adhesion

## Abstract

For *Entamoeba histolytica* to establish an infection, it must employ several mechanisms of pathogenicity to produce and secrete virulence factors that allow the parasite to adhere to and finally colonize and invade the host. However, in the intestinal epithelium, trophozoites (amoebae) encounter lactoferrin (Lf), a glycoprotein of the first line of defense, together with immunoglobulins and other molecules. We previously reported that iron-free bovine Lf (bLf) could kill amoebae both in vitro and in animal models of intestinal and hepatic amoebiasis. In this work, selected pathogenic mechanisms were evaluated in trophozoites by exposing cultures to sublethal concentrations of bLf to determine which amoebic functions could be altered. At a sublethal bLf concentration, this glycoprotein was removed from the trophozoite. In the presence of erythrocytes, bLf colocalized with erythrocytes at the capping site; this was observed by confocal microscopy of living cells. In addition, the erythrophagocytosis rate, proteolytic activity, adhesion, and cytotoxic properties towards Caco2 colonic cancer cells were reduced in the presence of bLf. Lactoferrin could be a particularly important protein that naturally protects colonic epithelial cells from *E. histolytica* infection.

## 1. Introduction

*Entamoeba histolytica* is a parasitic protozoan (amoeba) that infects the large human intestine and causes amoebiasis in approximately 10% of the global population, resulting in 40,000 to 100,000 deaths per year. Amoebiasis is an infection of great importance since it is the second leading cause of death from intestinal infection in children. This infection can be asymptomatic; nevertheless, some infected individuals may experience severe complications, as the parasite can invade intestinal tissue, resulting in pain, colitis, and dysentery. In some cases, amoebae can invade liver, lung, or brain tissues [1,2,3].

Direct adhesion of amoebae to host cells mediated by the Gal/GalNAc lectin has been reported [4]. Invasive disease occurs when trophozoites in the colon penetrate and cross the intestinal protective mucus layer. Amoebic infection requires degradation of the mucus and the extracellular matrix by parasite enzymes, which is critical for tissue invasion and evasion of the host immune response [5]. Amoebic proteases degrade immunoglobulins, defensins, mucin, villin, and cytokines and induce apoptosis in epithelial cells [6,7]. Amoeba trophozoites also produce amoebapores, a family of ion channel-forming peptides that are spontaneously incorporated into cellular membranes, causing a very rapid collapse of membrane integrity and resulting in contact-dependent cytolysis [8,9,10]. In invasive infection, amoebae migrate towards epithelial cells to phagocytize them, including both live and apoptotic cells. The damage continues with the activation of immune cells, phagocytosis, and ultimately, the development of an inflammatory response caused mainly by neutrophils. All these processes result in severe injury to the intestinal mucosal surface, which is ultimately exposed to parasites that multiply and may disseminate via the portal vein to invade other organs [11,12,13]. Parasites can also phagocytose leukocytes and erythrocytes and can perform trogocytosis, a process in which they ingest small portions of the host cell, causing death [2,5]. Adhesion to cells, cytotoxicity, phagocytosis, and proteolytic activity are key mechanisms employed by *E. histolytica* for infection and invasion of the host.

Lactoferrin (Lf) is a glycoprotein of the mammalian innate immune system that is found mainly in colostrum and milk and in minor quantities in tears, saliva, nasal and bronchial mucosae, and seminal and gastrointestinal fluids. Lf is also stored in the secondary granules of polymorphonuclear leukocytes and is secreted at sites of infection. It is considered a nutraceutical molecule owing to its important role as an immunomodulator and antioxidant agent. In addition, as a host defense molecule, Lf is important as an antiviral, antibacterial, antibiofilm, antifungal, and antiparasitic agent. Other notable functions include its anti-inflammatory and anticancer activities. Human and bovine Lf share 69% of their protein sequence [14,15,16].

Normally, when it is secreted, Lf in humans is not completely saturated with iron and is referred to as native Lf (15–20% iron). The saturation level depends on the diet and the iron level in the body [17]. As an antimicrobial agent, Lf is microbiostatic because of its ability to bind ferric iron, thereby preventing pathogens from obtaining iron for their growth, which is an essential nutrient for almost all living organisms; invasive amoebae are no exception. Lf acts as a bactericidal agent, and its activity leads to membrane and cell wall destabilization, destroying the bacterial cells [18]. Studies on the effects of Lf on several protozoa have been published [19]. As observed in bacterial cells, in protozoan parasites, Lf inhibits parasite growth by limiting access to iron and by competing for the iron-binding site of the pathogen. When Lf is bound to the parasite membrane, it causes membrane destabilization, as described in *Giardia lamblia*, the most common parasite infecting children, where it inhibits its growth and cellular differentiation. Lf can kill this protozoan by causing morphological changes in the plasmalemma, internal membrane, cytoskeleton, and lysosomes [20,21,22]; however, the addition of iron prevents parasites from dying [23].

In some intracellular pathogens, the antimicrobial mechanism of Lf acts primarily through host macrophages, promoting their activation to phagocytize and inhibiting their growth, as was observed in mouse peritoneal macrophages infected with *Toxoplasma gondii*, *Trypanosoma cruzi* and *Leishmania* spp., with increased free radical production, phagocytosis, and signaling pathways of the Toll-like receptor (TLR) and decreased expression of the multidrug resistance 1 (MDR1) gene [24,25,26]. In summary, the activity of Lf against microbial pathogens includes membrane destabilization and the targeting of cell invasion processes.

We reported that human holo-bLf (iron-charged bovine Lf, >95% iron) is used by *E. histolytica* trophozoites as the sole iron source in vitro and is found inside endosomes [27]. In contrast, human and bovine apo-Lf (0–5% iron) molecules bind to the amoeba membrane and exhibit antiamoebic activity within 30 min of binding, leading to parasite collapse [28]. We subsequently confirmed the amoebicidal effect of apo-Lf in a hamster model of hepatic abscess and a mouse model of intestinal infection [29,30]. In addition, we observed a synergistic effect of bLf and metronidazole, the selected drug used to treat amoebiasis [31]. The mechanisms by which Lf inhibits amoeba growth and affects its pathogenic capacity are not completely understood. This work aims to provide insight into the effects of low-iron Lf on several *E. histolytica* pathogenicity mechanisms, such as adhesion, cytotoxicity, erythrophagocytosis, and protease secretion. The in vitro effectiveness of Lf in killing protozoa supports the possibility of using it either alone or in combination with the usual treatments as a therapeutic agent for several parasitic infections that affect humans, including concomitant infections.

## 2. Results

### 2.1. Effect of bLf on the Viability of Trophozoites of Entamoeba histolytica

**Figure 1:**
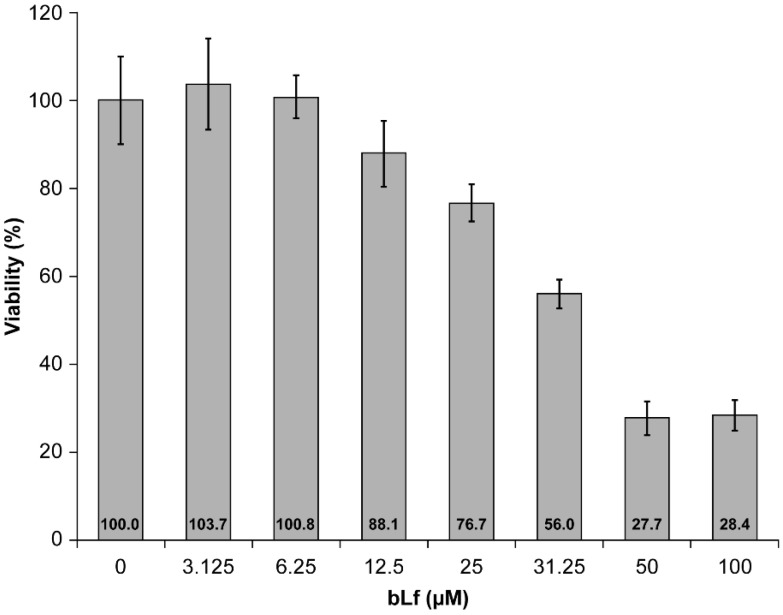
Viability of *Entamoeba histolytica* trophozoites incubated with different concentrations of bLf. After 24 h of culture with bLf, the viability was measured by the MTT assay and compared with that of amoebae incubated without bLf. Data represent the mean ± SDs of three independent experiments. Statistical significance relative to the untreated control was determined using Student’s *t*-test. (*p* < 0.001 for 12.5 µM (*p* = 0.00094), non-significant for 3.125 and 6.25 µM). The concentration of 12.5 µM was established as sublethal based on maintaining trophozoite viability above 80%.

Figure 1 shows that the number of live amoebae decreased with increasing bLf concentration. Amoebae maintained normal growth at low bLf concentrations (6.25 and 12.5 µM), whereas at 25 µM, the viability was approximately 77%. The highest concentrations (50 and 100 µM) did not reduce the viability to less than 27%; thus, bLf affects amoebic growth. To observe the effects of bLf on the mechanisms underlying the pathogenicity of this parasite, in subsequent experiments, 12.5 µM Lf (a sublethal concentration of 88% viability) was used.

### 2.2. Adhesion to Caco2 Cells

**Figure 2:**
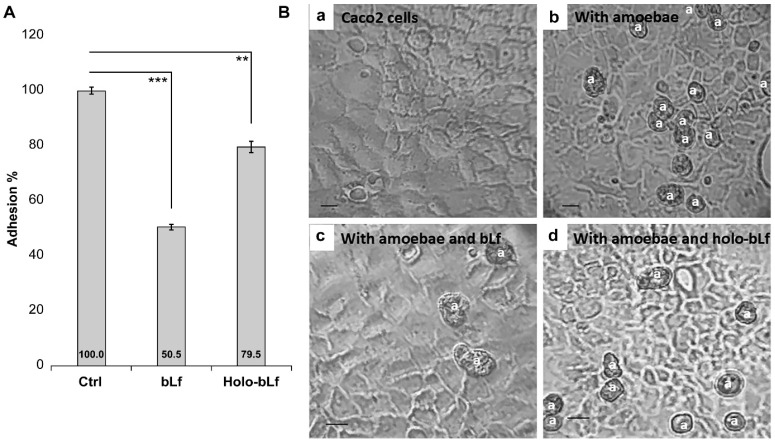
Adhesion of amoebae to Caco-2 cells in confluent cultures. Amoebae treated with bLf were incubated with previously fixed Caco-2 cells, and the nonadherent cells were counted. (**A**) Percentage of attached amoebae; (**B**) Microscopy of Caco-2 cells without amoebae (**a**); with amoebae without bLf (**b**); with amoebae treated for 1 h with bLf (**c**); with amoebae treated for 1 h with holo-bLf (**d**). Amoebae are marked with a white “a.” Scale bar, 25 µm. Data represent the mean ± SD of three independent experiments. Statistical significance relative to the untreated control was determined using Student’s *t*-test, ** *p* < 0.01, and *** *p* < 0.001.

Adhesion of pathogens to target cells is the first step in colonization and invasion. To evaluate whether bLf affects amoebic adhesion to cells, we tested this pathogenic mechanism in Caco2 cultures. Amoebae previously incubated for 1 h with bLf were added to fixed confluent cell cultures. Afterwards, culture medium supernatant (SN) with nonadherent amoebae was obtained, and the difference between the initial number of amoebae added and the number of nonadherent amoebae was determined. The results are shown in Figure 2A, where amoebae treated with bLf presented the lowest adhesion rate (50%) compared with those without bLf or with holo-bLf. A confluent monolayer of Caco-2 cells is shown in Figure 2B. Following the addition of bLf-treated amoebae, the number of observed parasites was markedly lower than that in samples involving untreated amoebae or those treated with holo-bLf. These results suggest that bLf protects cells from amoebic adhesion.

### 2.3. Confocal Microscopy of Trophozoites Incubated with bLf

**Figure 3:**
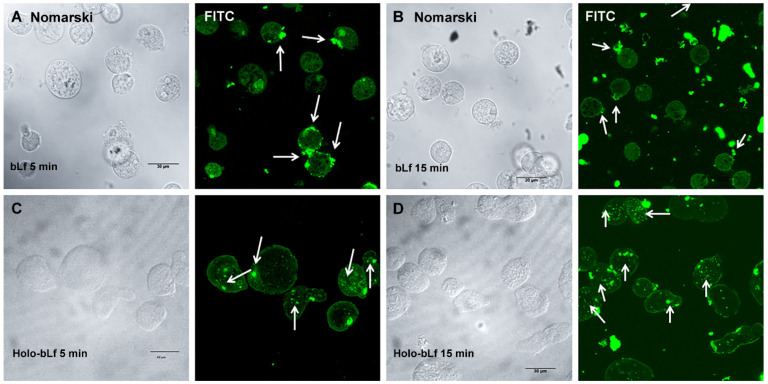
Confocal microscopy of *Entamoeba histolytica* trophozoites incubated with FITC-bLf and FITC-holo-bLf as a control. Trophozoites were observed under Nomarski optics and fluorescence illumination. (**A**) Trophozoites were incubated for 5 min with FITC-bLf; fluorescence was observed on the membrane as small patches. (**B**) At 15 min, the patches coalesced to form a large patch that was released into the extracellular medium, as fluorescent material was observed outside the cell. In contrast, in the presence of holo-bLf, a protein that is normally endocytosed and used as an iron source by the parasite, the protein is present in vesicles inside the cell (**C**,**D**). The procedures were repeated three times each. Scale bar 30 µm; white arrows point to the concentration of bLf; On the membrane A and B, inside the cell in C and D.

To determine the effect of a sublethal concentration of bLf in trophozoites, they were incubated at 12.5 µM to maintain viability above 80%. As observed in Figure 3, at the beginning of the interaction (5 min) between the amoebae and bLf, this glycoprotein was distributed along the cell surface in patches (Figure 3A), and the amoebae appeared rounded in comparison to the holo-bLf, which was internalized by the amoebae as early as this time point (Figure 3C). After 15 min (Figure 3D), holo-bLf was distributed in vesicles inside the cytoplasm of all the amoebae. However, at 15 min with bLf, this protein was concentrated in some areas of the plasma membrane, and bLf was apparently removed from the amoebae (Figure 3B).

### 2.4. The Effect of bLf on erythrophagocytosis

**Figure 4:**
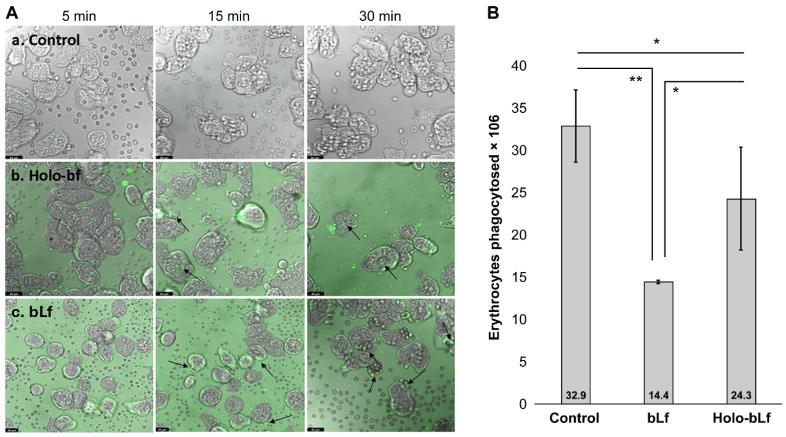
Erythrophagocytosis. (**A**) *E. histolytica* trophozoites were incubated with FITC-bLf or FITC-holo-bLf for 1 h, incubated with erythrocytes (200:1), and observed by live-cell confocal laser microscopy: (**a**) amoebae incubated with erythrocytes; (**b**) amoebae incubated with FITC-holo-bLf; and (**c**) amoebae incubated with bLf. Erythrocytes are shown accumulated at one pole of the amoeba, in the same site where bLf is localized, and were subsequently detached. The arrows point to the sites where holo-bLf was internalized in roud shape structures in Figure 4b and where bLf released in Figure 4c. Scale bar 30 µm. (**B**) The bar graph shows a 56% decrease in the number of ingested erythrocytes when amoebae were incubated with bLf (14.4 erythrocytes) compared with trophozoites without bLf (24.3 erythrocytes). Holo-bLf reduced erythrophagocytosis by only 25%. Data represent the mean ± SD of three independent experiments. Statistical significance was determined using Student’s *t*-test. * *p* < 0.05, and ** *p* < 0.01.

To observe the effect of bLf on amoeba erythrophagocytosis, we used confocal laser microscopy of live cells to analyze the interaction between amoebae and erythrocytes. When amoebae were previously treated with bLf, they appeared rounded, bLf was observed surrounding the membrane (not in patches), and the number of erythrocytes decreased to 15–18. In addition, as described previously [32,33] after 15 min of interaction, some erythrocytes accumulated at one pole outside the parasite membrane (Figure 4A(a)). After 30 min of interaction, bLf-treated amoebae presented bLf localized at the same pole where erythrocytes accumulated (Figure 4A(c)). The hemoglobin content from phagocytosed erythrocytes in amoebae treated with bLf was quantified and was found to be approximately 15 erythrocytes/amoeba (Figure 4B). The amoebae phagocytized erythrocytes in a time-dependent manner (approximately 30 erythrocytes per amoeba at 30 min); the same result was obtained when holo-bLf was used. Confocal microscopy of live amoebae shows that bLf was released when erythrocytes in live amoebae were concentrated at one pole of the cell. These results suggest that bLf but not holo-bLf affects amoebic erythrophagocytosis.

### 2.5. Cytotoxicity to Caco2 Cells

**Figure 5:**
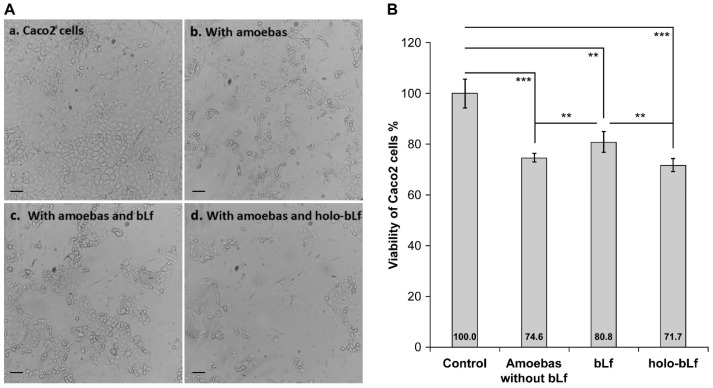
Cytotoxic effect of amoebae treated with bLf on Caco-2 cells. (**A**) Control cells without treatment (**a**), cells infected with untreated amoebae (**b**), cells infected with amoebae treated with bLf (**c**), and cells infected with amoebae treated with holo-bLf (**d**). Cells were fixed, stained with methylene blue, and observed by light microscopy. Bar, 50 µm. (**B**) The extracted dye was measured at 660 nm, and the percentage of viable cells was determined. Data represent the mean ± SD of three independent experiments. Statistical significance relative to the untreated control was determined using Student’s *t*-test. ** *p* < 0.01, and *** *p* < 0.001.

To determine whether bLf affects the cytotoxicity of *E. histolytica* to intestinal cells, we incubated amoebae treated with bLf with confluent Caco2 cells at a 1:2 (cell: amoeba) ratio for 1 h. The cytotoxic effect was slightly reduced when amoebae were treated with bLf (Figure 5A(b)); however, bLf partially protected against the destruction of cell confluence (Figure 5A(c)) compared with amoebae without bLf (Figure 5A(a,b)) or with holo-bLf (Figure 5A(d)).

### 2.6. Lactoferrin Degradation by Entamoeba histolytica Proteases

**Figure 6:**
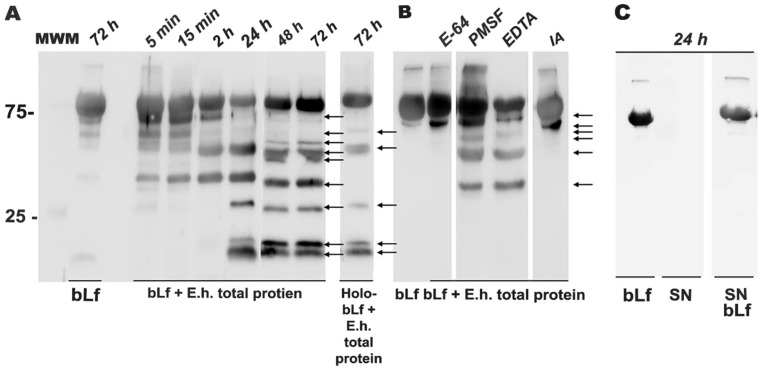
Lactoferrin is cleaved by native amoebic proteins. Amoebic total protein extracts (**A**) were incubated with bLf for different durations and electrophoresed on 12% SDS–PAGE gels. bLf degradation bands were identified with anti-bLf antiserum after protein transfer to nitrocellulose membranes. (**B**) Total protein extracts with bLf were incubated with protease inhibitors: E-64, trans-epoxysuccinyl-L-leucylamido (4-guanidino) butane; PMSF, phenylmethylsulfonyl fluoride; EDTA, ethylenediaminetetraacetic acid; IA, iodoacetamide. Only cysteine protease inhibitors prevented bLf degradation at 24 h. (**C**) Supernatants from the culture media (SN), previously centrifuged to remove amoebae and cell debris, were incubated with bLf for 24 h. Proteases in the SNs were unable to degrade bLf. Black arrows mark degradation bands of bLf and holo-bLf. The procedures were repeated three times each.

An important pathogenic mechanism of *E. histolytica* is the production and secretion of proteases. To test whether a sublethal concentration of bLf affects the activity or secretion of amoebic proteases, total protein from amoeba cells (extracts) and from SNs was obtained from amoebae incubated with or without bLf or with holo-bLf. Amoebic proteins were incubated with bLf or holo-Lf for different durations, and Western blotting was performed using anti-bLf serum to identify any degradation bands. The amoebic proteolytic activity of the total extracts against bLf as early as 5 min is shown in Figure 6A, with more extensive degradation occurring after 48 and 72 h of incubation. A similar effect was observed with holo-bLf (only 72 h is shown). As shown in Figure 6B, bLf and amoeba total protein were incubated in the presence of protease inhibitors (10 µM E-64, 12 mM PMSF, 30 mM EDTA, and 50 µM iodoacetamide); only cysteine protease inhibitors prevented bLf degradation at 24 h. There were no differences between the amoebae incubated or not with bLf before protein extraction. However, when the SN from amoeba treated with bLf was tested, it did not show any degradation band for bLf at any of the tested times. These results suggest that although amoebic proteases are produced (protein quantity in SNs does not show differences between samples), they could not be active, perhaps due to a competitive inhibition effect for bLf.

### 2.7. Amoebic Proteolytic Activity Assessed by Zymography and Azocoll Cleavage

**Figure 7:**
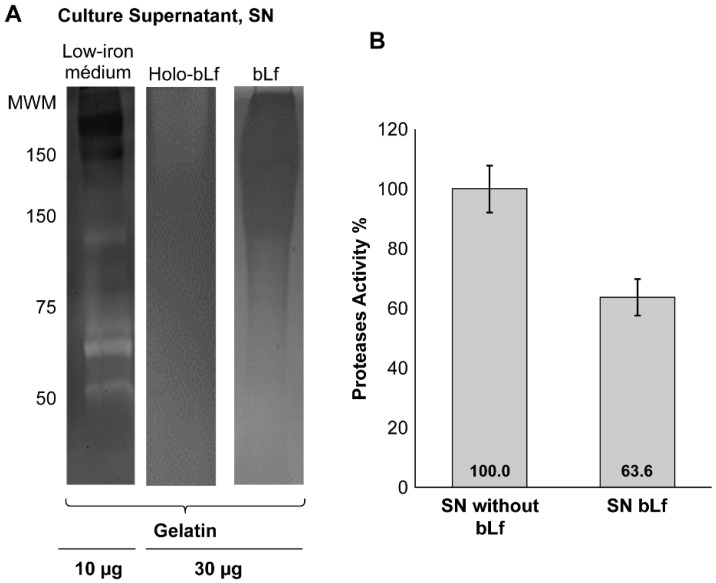
Secretion of proteases by *Entamoeba histolytica* trophozoites incubated with bLf. (**A**) Electrophoresis of culture supernatant (SNs) proteins on gels copolymerized with gelatin from amoebae grown in the indicated media. (**B**) Quantification of proteolytic activity using azocoll as a substrate. In amoebae cultured in the presence of bLf, the proteolytic activity against azocoll in the SN decreased by approximately 37%, and the activity was completely abolished in the presence of PHMB (a cysteine protease inhibitor, material intended for publication). Data represent the mean ± SDs of three independent experiments. Statistical significance relative to the untreated control was determined using Student’s *t*-test. The procedures were repeated three times each.

Gels copolymerized with gelatin as a standard substrate for proteolytic degradation were used. Proteases were present in the SNs of amoebae grown in low-iron medium (Figure 7A). Interestingly, in the case of the SNs of amoebae grown with bLf or holo-bLf, no activity was detected even when threefold the sample quantity was loaded. When the proteolytic activity of bLf-treated SNs was quantified using azocoll as a substrate (Figure 7B), it was observed to be 63.6% relative to the low-iron control. When PHMB was used, the activity was not detected.

These results suggest that bLf has a protective effect through the inhibition of proteolytic activity, perhaps by binding directly to the amoebic proteases or inhibiting the secretion of proteolytic enzymes into the SNs.

## 3. Discussion

The ability of *Entamoeba histolytica* to establish itself in its niche ultimately determines whether the infection persists or if the host successfully clears the parasite. The ability of trophozoites to colonize the host depends on their pathogenic mechanisms and on the production and secretion of virulence factors, which provide the trophozoites with an enhanced ability to survive the attack of immune cells. Adhesion to intestinal cells, erythrophagocytosis, cytotoxicity, and protease activity are key factors in amoebic infections. Therefore, the success of a parasite depends on its ability to colonize the host or to circumvent innate defense, such as Lf. As a multifunctional glycoprotein that is ubiquitous in mucosal secretions, Lf serves as a primary barrier against pathogens while maintaining intestinal homeostasis. Lf has beneficial antipathogenic properties, helping to maintain the integrity of the intestinal barrier and protect it against pathogens [34].

Low-iron Lf can reach the intestine either as a whole molecule or as bioactive fragments such as lactoferricin and lactoferrampin [15], interacting directly with *E. histolytica* [30]. In this context, understanding how Lf affects amoebae in the intestine helps to prevent colonization. This interaction was studied by using sublethal concentrations of bLf, since high concentrations of bLf could kill amoebae within a short period of time [28].

For the colonization process, *E. histolytica* trophozoites must invade the intestinal mucosa, confronting the mucus barrier above a single layer of epithelial cells. These cells are protected from bacterial and amoebic infections through IFN-Ɣ, mucosal IgA [35], and endogenous Lf because of their presence in exocrine secretions, which enhances mucosal immunity [17]. In this work, we demonstrate that the ability of amoebae grown in the presence of sublethal concentrations of bLf to adhere to Caco2 cells is significantly impaired (50.5%). This reduction in adhesion is consistent with previous reports indicating that bLf disrupts membrane phospholipids [31] and may therefore interfere with the organization of the EhGal/GalNAc lectin and the cytoskeleton, both of which are essential for host cell contact.

In the case of holo–bLf, it exerts an inhibitory effect, reducing *E. histolytica* adhesion to approximately 79.5% compared to the untreated control. A direct statistical comparison between the two treatment groups confirmed that the superior efficacy of bLf over holo–bLf is statistically significant (*p* = 0.083). Since trophozoites actively bind and endocytose the iron-saturated holoprotein to fulfill their metabolic iron demands, the phenotypic impairment is less severe than that induced by iron-free bLf. Consequently, a higher number of viable adhering amoebae is recovered following holo-bLf incubation compared to the bLf group, highlighting how iron availability distinctively modulates surface virulence factor deployment.

When amoebae incubated with bLf were observed by confocal microscopy, bLf was found on the cytoplasmic membrane and, within a few minutes, was observed to be expelled outside the trophozoites. When bLf is iron-saturated (holo–bLf), it is observed inside amoebae in vesicles, as reported previously [28]. Therefore, trophozoites incubated with low-iron bLf do not allow bLf to penetrate inside the cell, unlike holo–bLf (iron-saturated protein), which serves as an iron source for the amoeba.

Erythrophagocytosis is a well-described virulence factor and is a hallmark of *E. histolytica* virulence [36,37,38]. Erythrocytes were phagocytosed actively by amoebae. Only after several minutes of interaction does phagocytosis become polarized to a single pole of the parasite, resulting in a significant accumulation of erythrocytes at one pole of the trophozoites in the uroid region. In *E. histolytica*, capping and the subsequent shedding of the uroid serve as an immune evasion mechanism that removes bound host molecules [33,37,39,40]. The capping and release of bLf was shown to be actin-independent in round-shaped structures. This loss of membrane could lead to the damage of associated material, likely depleting essential adhesion proteins and receptors, thereby reducing the parasite’s invasive capacity.

On the other hand, the presence of bLf diminished by nearly 50% the number of erythrocytes phagocytosed, compared to amoebae that were not treated or that were treated with holo–bLf. Similar results were obtained by biochemically measuring hemoglobin content. Furthermore, the colocalization of FITC-bLf and erythrocytes, along with their inability to be effectively internalized, suggests that bLf either sterically blocks binding or induces an early redistribution of the phagocytic machinery toward one zone of the parasite for excretion. As a result, this process removes the parasite’s molecular tools essential for host tissue invasion—such as adhesion, erythrophagocytosis, and cytotoxicity—processes in which cytoskeletal participation is well established [3,41]. Whether bLf interacts directly or indirectly with related proteins remains a subject for future research.

This finding indicates that even the iron-saturated form of bovine lactoferrin maintains distinct bioactivity against *E. histolytica*. This partial inhibition may be attributed to a metabolic adaptation phase during the coincubation of trophozoites, holo-bLf, and erythrocytes. Because *E. histolytica* needs to fulfill its physiological iron requirements, the presence of holo–bLf provides an accessible, alternative iron source aside from erythrophagocytosis. The capacity of holo–bLf to maintain growth has been previously reported [27]. Therefore, the bioactivity of Lf plays a dual role: bLf regulates surface-associated pathogen virulence factors, while holo–bLf is used for iron acquisition.

Interestingly, elucidation of the relationship between bLf and the cytotoxicity of *E. histolytica* will provide new insight into the pathogenic process leading to amoebiasis. The ability to adhere to human host cells is necessary for human host entry and the subsequent cytotoxicity process [3]. In this case, the cytotoxicity of amoebae incubated with bLf to Caco2 cells was reduced by almost 20%. Although amoebae can be defective in adhesion and phagocytosis, they remain capable of disrupting the monolayer of intestinal cells. This is because *E. histolytica* adhesion assays were conducted using fixed Caco-2 monolayers, which preserve surface binding sites but abolish dynamic cellular signaling and membrane integrity. In contrast, the cytotoxicity assay utilizes a live culture system where *E. histolytica* can deploy its multi-factorial and redundant pathogenic arsenal. *E. histolytica* employs independent cytotoxic mechanisms, such as the secretion of cysteine proteases, amoebapores, and trogocytosis. Amoebapores, which are cytotoxic factors, are ion-channel-forming proteins that spontaneously incorporate into cellular membranes; cysteine proteases affect host tissues and cells at the site of infection, enhancing virulence [8,9]; and trogocytosis is a mechanism by which the amoeba ingests fragments of the host cell membrane, leading to host cell death. Normally, this occurs when an amoeba infects intestinal cells [5].

Cysteine proteases (CPs), which are pivotal virulence factors in *Entamoeba histolytica* that act either intracellularly or through secretion to disrupt host tissues and modulate immune responses [7], increase pathogenicity by degrading essential host defenses, including immunoglobulins, complement factors, and cytokines—while simultaneously dismantling the extracellular matrix (ECM) [2]. Contact-independent host cell death caused by *E. histolytica* is driven primarily by secretory CPs that degrade ECM components such as elastin, collagen, and fibronectin. Since the survival of epithelial cells depends on stable adhesion, the enzymatic degradation of the ECM triggers anoikis—a specialized form of apoptosis—resulting from the loss of cell–matrix interactions [42,43].

In this work, the data confirmed that *E. histolytica* proteases in the total protein extracted remained active in the presence of bLf, as evidenced by the degradation of bLf (Figure 6) and fibronectin (the results with fibronectin are Material intended for publication). Under these natural conditions, bLf is degraded because proteases operate within a complex environment affected by factors such as enzyme concentration, cofactors, ion concentrations, or the concerted activity of two or more proteases. To determine whether the secreted proteases were active, the SNs of the culture media were electrophoresed on an SDS–protease gel with gelatin. The substrate was not degraded, even though the protein quantity measured in the SN was not different from that of the amoebae without bLf (the amount of secreted proteins was equal) (Figure 7A); the substrate was not degraded even when the protein quantity was increased. These findings could be related to the effect of bLf at the membrane level [28], and protease activity could subsequently be affected because of the inactivation of the protease itself caused by bLf binding.

Azocoll assays were performed to quantify the proteolytic activity of the SNs (collagen; Figure 7B). Under these conditions, the activity of the SNs decreased by approximately 40%. This result confirms the inhibitory effect of bLf on protease activity; nevertheless, the remaining activity suggests the presence of other proteases or an incomplete inhibition of the total proteolytic profile by bLf. Therefore, it is evident that other cytotoxic mechanisms could be facilitating the membrane disruption independently of protease activity, persisting even when parasite adhesion is impaired, both produced by the bLf action.

Our findings in Caco-2 cells align with this mechanism, as we observed that *E. histolytica*-secreted CPs induce significant cell detachment and destruction. This proteolytic effect slightly diminished when the amoebae were treated with bLf. Interestingly, while total amoebic proteins incubated with bLf under native conditions showed complete degradation of bLf itself, zymography assays revealed the complete disappearance of the proteolytic activity bands in the presence of bLf.

Similarly, a total loss of activity was observed by gel electrophoresis with gelatin, bLf, collagen, and holo–bLf as substrates (Material intended for publication), suggesting that bLf has a potent inhibitory effect on amoebic proteases. This inhibition could occur mainly by binding to high-affinity protease active sites or interfering with the stability of high-molecular-weight protease complexes in synergy with other virulence factors, such as adhesins. Given that previous studies have shown that amoebic proteases function within multiprotein complexes [42,44], bLf may disrupt the synergy between CPs and other virulence factors. Further research is needed to determine whether bLf or its derived peptides directly block specific protease isoforms or cause structural dissociation of these functional complexes. These findings reinforce the role of bLf as a promising therapeutic agent capable of tempering *E. histolytica* virulence.

In conclusion, although *E. histolytica* can degrade bLf via cysteine proteases, the initial interaction with sublethal concentrations of this protein triggers a massive redistribution of membrane components. This reduces the ability of the parasite to adhere to and ingest host cells, providing new insights into how Lf could contribute to the early control of amoebiasis. We can summarize the effects of bLf as follows: (1) bLf inhibits parasite growth by sequestering the iron needed for survival; (2) a sublethal concentration of bLf induces erythrocyte accumulation at one zone of the parasite; (3) bLf binds to the parasite plasma membrane and is subsequently expelled out of the cell to the extracellular media, affecting proteins of its membrane; (4) the structure of the membrane and its properties are related to the virulence of the amoebae and their invasive capacity, which affects membrane functions, mainly adhesion and phagocytosis; and (5) protease SNs activity may be affected as a consequence of bLf binding directly to the proteases.

Bovine lactoferrin impairs the virulence of *E. histolytica* by restructuring its membrane components and inhibiting adherence and erythrophagocytosis. Membrane lipid and/or protein redistribution could prevent the parasite from ingesting host cells. Nevertheless, further studies are needed to fully elucidate the specific effects of bLf on *E. histolytica* trophozoites.

## 4. Materials and Methods

### 4.1. Cultures of Trophozoites and the Cell Line

Amoebae were grown in axenic BI-S-33 medium (Cat. 2001-E Dibico, Mexico City, Mexico), supplemented with 16% bovine serum (Cat. SU-S40 MicroLab, Mexico City, Mexico) at 37 °C; this medium contains ammonium-ferric citrate as the main iron source (approx. 100 µM total iron). All experiments were performed with amoebae cultured for 48 h and then incubated for 24 h in medium pretreated with Chelex 100 resin (Cat. 142-2842 Bio-Rad, Lab. Inc., Hercules, CA, USA) to remove free iron; the medium was sterilized, and bLf (or holo-bLf as a control) was added after sterilization by filtration through a 0.22 µm membrane. Then, medium supplemented with 8% bovine serum was added for the assays. Bovine Lf was obtained from NutriScience Innovations, Milford, CT, USA). To obtain the amoebae, the tubes were placed in an ice bath for 15 min and then centrifuged at 500× *g* for 5 min. In this work, we used holo-bLf (100% iron) as a control and bLf (14.1%) as the low-iron bovine Lf for the assays.

The human colorectal epithelial cancer cell line Caco2 (ATCC HTB-37) was cultured in DMEM (Cat. 11965092 Gibco, Grand Island, NY, USA) supplemented with 10% fetal bovine serum (Cat. 16000044 Gibco, Grand Island, NY, USA), 100 μg/mL streptomycin, 100 U/mL penicillin, and 2 mM glutamine (Cat. 12100046 Gibco, Grand Island, NY, USA) at 37 °C in an atmosphere with 5% CO_2_.

### 4.2. Rabbit Anti-bLf Antibody Production

For antibody production, a New Zealand female rabbit weighing 2.5 kg was subcutaneously inoculated with a bLf band emulsified with aluminum hydroxide and excised from a 10% SDS–PAGE gel stained with Coomassie blue. The rabbit received three immunizations of 75 µg of bLf at 15-day intervals. To corroborate the production of antibodies, Western blot assays were performed. After electrophoretic separation of Lf by 10% SDS–PAGE, the protein was transferred to a nitrocellulose membrane (Cat. 162-01-15 Bio-Rad Lab. Inc., H) for 1 h at 400 mA. The membrane was blocked with bovine serum albumin (Cat. A2153 Sigma–Aldrich, Inc., St. Louis, MO, USA) overnight at 4 °C and then incubated for 2 h with rabbit anti-bLf serum (1:1000). The membrane was subsequently washed three times with PBS–Tween (Cat. P9416 Sigma–Aldrich Inc., St. Louis, MO, USA) (0.05%) and incubated with an anti-rabbit-HRP secondary antibody (1:5000; A-0545; Sigma–Aldrich Inc., St. Louis, MO, USA) for 1 h. The membrane was washed three times, after which the signal was developed with a luminol kit (Cat. WBKLS0100, Millipore, Burlington, MA, USA). Once an adequate antibody titer was confirmed by ELISA, terminal blood collection was performed under deep anesthesia to maximize serum yield [45,46].The rabbit used in this study was handled according to the protocol 338-22 approved on 13 February 2023 by the Institutional Committee (IACUC) of Centro de Investigación y de Estudios Avanzados (Cinvestav-IPN), Mexico. Our institution fulfilled the technical specifications for the reproduction, care, and use of laboratory animals and is certified by federal law (NOM-062-ZOO-1999).

### 4.3. Effect of bLf on the Viability of E. histolytica Trophozoites

In previous works [29,30,31,47], bovine Lf from Morinaga Milk Co. (Minato-Ku Tokyo, Japan) was used at concentrations between 12.5 and 100 µM, resulting in amoebae viability of 10% at the highest concentration. Owing to the fact that the bovine Lf used in this work was obtained from a different company (NutriScience Innovations, Milford, CT, USA), measuring the amoeba viability was necessary. In this case, the iron concentration of bLf was 14.1%. Viability was measured using a modified MTT assay, according to the method reported by Díaz-Godínez (2019) [48]. Briefly, 2 × 10^4^ amoebae/well were placed in 96-well plates in iron-free medium supplemented with 8% bovine serum and bLf at different concentrations (6–200 µM). After incubation for 24 h at 37 °C, MTT (1 mg/mL; Cat. M5655 Sigma–Aldrich Inc., St. Louis, MO, USA) was added, and the mixture was incubated for 1 h at 37 °C in the dark. Afterwards, 15% SDS (Cat. 161-0302, Bio-Rad Lab. Inc., Hercules, CA, USA) in 0.01 N HCl (Cat. 7647-01-0 Merck, Darmstadt, Germany) was added at 60 °C. The absorbance was measured at 595 nm. Three independent experiments were performed, each in triplicate, and the results are expressed as the means ± SDs.

### 4.4. Adhesion of E. histolytica to Caco2 Cells

For the adhesion assay of *E. histolytica* to Caco2 cells, amoebae were added to cells in 12-well cell culture plates at a confluency of 4 × 10^4^ cells/well [49]. The cells were subsequently washed twice with sterile PBS (pH 7.4) and fixed for 30 min with 2% paraformaldehyde (Cat. 30525-89-4 Electron Microscopy Sciences, EMS; Hemlock Road, Morgantown, PA, USA) in PBS at room temperature. The samples were thoroughly rinsed with PBS. Afterwards, the amoebae were incubated for 1 h at 37 °C in iron-free medium with or without bLf (12.5 µM), added at a 1:2 (cell: amoebae) ratio, and incubated for 30 min at 37 °C. Finally, the supernatant was removed, the cells were washed twice with PBS, and the weakly adherent and nonadherent trophozoites were counted in a Neubauer chamber. The number of adherent trophozoites was calculated as the difference between the initial number of amoebae added and the number of nonadherent trophozoites.

### 4.5. Confocal Microscopy of Fixed Trophozoites Incubated with bLf

Amoebae (2 × 10^5^ parasites/mL) were incubated on slides in iron-free medium for 20 min at 37 °C. Then, FITC-holo-bLf (a positive control for endocytosis) or FITC-bLf was added (at a sublethal concentration of 12.5 µM) and incubated at 37 °C. At 0, 5, 7.5, 10, 15, and 30 min, the amoebae were fixed with 2% paraformaldehyde (Electron Microscopy Sciences, EMS; Hemlock Road, Morgantown, PA, USA) in PBS at room temperature, washed with PBS, mounted with glycerol: PBS (1:10; Cat. G7893 Sigma–Aldrich Inc., St. Louis, MO, USA), and observed by confocal laser microscopy (Leica Microsystems, Wetzlar, Germany.

### 4.6. Erythrophagocytosis

Human erythrocytes from the peripheral blood of healthy women (O group, Rh-positive) were used throughout the experiments. Erythrocytes were obtained in PBS-EDTA (EDTA, ethylenediaminetetraacetic acid, Cat. E9884; Sigma–Aldrich Inc., St. Louis, MO, USA) and washed three times to remove white blood cells and plasma proteins. Erythrocytes were counted and used at a 1:200 (trophozoite:erythrocyte) ratio for quantitative erythrophagocytosis assays. To observe the erythrophagocytosis process by confocal laser microscopy (Leica Microsystem, Wetzlar, Germany) of living amoebae, erythrocytes were added to amoebae previously bound to a confocal dish plate (Cat. 101350, SPL Life Sciences Co., Ltd., Pocheon-si, Gyeonggi-do, South Korea) (1 amoeba: 30 erythrocytes) and incubated with or without FITC-bLf or FITC-holo-bLf. The interaction was carried out for 40–120 min at 37 °C, and images were captured every 20 s (data are summarized in Figure 4). Three independent experiments were performed, each in triplicate.

To determine the effect of bLf on erythrophagocytosis, we preincubated 1 × 10^6^ amoebae for 1 h with or without bLf (12.5 µM) in tricine-saline solution (100 mL of 0.05 M Tricine Cat. T5816 Sigma–Aldrich (Inc., St. Louis, MO, USA); 900 mL of 0.85% NaCl solution; Cat. 3624-05 JT Baker, Phillipsburg, NJ, USA). Afterwards, 2 × 10^8^ erythrocytes were added and incubated for 30 min. At the end of the incubation, the amoebae and erythrocytes were centrifuged at 250× *g* for 3 min at 5 °C and resuspended in 1 mL of distilled water to induce lysis of non-ingested erythrocytes. Amoebae with ingested erythrocytes were resuspended in 1 mL of formic acid to release hemoglobin, and the absorbance was measured at 405 nm. Three independent experiments were performed, each in triplicate, and the results are expressed as the means ± SDs.

### 4.7. Cytotoxic Effect

Confluent Caco2 cells (4 × 10^4^ cells/well) were washed with DMEM (Cat. 11965092 Gibco, Grand Island, NY, USA) to remove fetal bovine serum (Cat. 16000044 Gibco Grand Island, NY, USA) and incubated for 1 h with trophozoites (8 × 10^4^/mL in DMEM), which were previously incubated with or without 12.5 µM bLf or holo-bLf for 1 h at 37 °C. The culture plate was cooled and carefully washed with cold PBS to remove nonadherent trophozoites. The cells remaining on the culture plate were fixed with 2% paraformaldehyde (Electron Microscopy Sciences, EMS; Hemlock Road, Morgantown, PA, USA) in PBS for 30 min at room temperature, washed with PBS, and stained with 0.1% methylene blue (Cat. M9140 Sigma–Aldrich Inc., St. Louis, MO, USA) in 0.1 M borate buffer, pH 8.7 (0.07 M boric acid Cat. 0084 JT Baker (Phillipsburg, NJ, USA); 0.01 M sodium borate Cat. 3568 JT Baker (Phillipsburg, NJ, USA), for 10 min, after which they were washed with 0.01 M borate buffer, and the dye was extracted with 0.1 M HCl at 37 °C for 30 min. Absorbance was measured at 660 nm using a Multiskan™ microplate photometer (Agilent Technologies, Inc. Santa Clara, CA, USA).

### 4.8. bLf Degradation by Amoebic Proteases Observed by Western Blotting

Total amoebic protein was obtained in Tris-CaCl_2_ (0.1 M Tris-base Cat. 77-86-1 Sigma–Aldrich (Inc., St. Louis, MO, USA); 10 mM CaCl_2_ Cat. 10035-04-8 Merck (Merck, Darmstadt, Germany); pH 7.0). Amoebic proteins (15 µg) were incubated with 15 µg of bLf or holo-bLf for different durations (5 and 15 min; 2, 24, 48, and 72 h). Afterwards, 2X sample buffer was added, and the mixture was boiled for 5 min with 5% β-mercaptoethanol (Cat. 161-0710 Bio-Rad Lab. Inc., Hercules, CA, USA). Samples (10 µg) and bLf or holo-bLf at 72 h were subjected to 12% SDS–PAGE and transferred to nitrocellulose membranes (Cat. 162-0115; Bio-Rad, Lab. Inc. Hercules, CA, USA), which were incubated with an anti-bLf antiserum (1:1000) and then with anti-rabbit secondary antibody (1:5000 Cat. A0545; Sigma–Aldrich Inc., St. Louis, MO, USA), and the signal was developed with a luminol kit (Cat. WBKLS0100; Millipore Burlington, MA, USA). The signal was photodocumented with an Odyssey XF Imager system (Agilent Technologies, Inc., Santa Clara, CA, USA).

### 4.9. Secreted Amoebic Proteolytic Activity on Gelatin, bLf, and Holo-bLf by Zymography

Amoebae were cultured in the presence or absence of bLf or holo-bLf for 24 h. Then, culture supernatants (SNs) were obtained. Proteins were precipitated with absolute alcohol (Cat. 64-17-5 Merck (Darmstadt, Germany) at −20 °C, for 3 h and electrophoresed in 7% gels copolymerized with 0.2% gelatin (Cat. 73865 Sigma–Aldrich Inc., St. Louis, MO, USA), bLf, or holo-bLf at 100 V. Before staining the gel with Coomassie blue dye (Cat. B7920 Sigma–Aldrich Inc., St. Louis, MO, USA), it was incubated with 2.5% Triton X-100 (Cat. X100 Sigma–Aldrich Inc., St. Louis, MO, USA) for 1 h and then in activation buffer containing 10 mM CaCl_2_ (Merck, Darmstadt, Germany, pH 7.0). In some experiments, the gels were incubated with the following protease inhibitors: 10 µM trans-epoxy succinyl-L-leucyl amido(4-guanidino) butane, E-64 (Cat. E3132 Sigma–Aldrich Inc., St. Louis, MO, USA); 12 mM phenylmethanesulfonylfluoride, PMSF (Cat. 78830 Sigma–Aldrich Inc., St. Louis, MO, USA); 30 mM EDTA (Sigma–Aldrich Inc., St. Louis, MO, USA); or 50 µM iodoacetamide (Cat. I6125 Sigma–Aldrich Inc., St. Louis, MO, USA).

To quantify proteolytic activity, we employed azocoll degradation (Cat. 194933 CALBIOCHEM, San Diego, CA, USA). Briefly, 375 µL of azocoll (1.5 mg/mL) was added to the same volume of each amoebic protein (SN) (10–50 µg/µL), the volume was adjusted to 375 µL with a buffer (pH 7.0), and the mixture was incubated at 37 °C for 2 h. Then, the mixture was centrifuged (10 min, 10,000× *g*), and the absorbance was read at 550 nm using a Multiskan™ microplate photometer (Agilent Technologies, Inc. Santa Clara, CA, USA). Protein degradation was proportional to the absorbance. The experiments were performed in triplicate.

### 4.10. Statistical Analysis

Statistical comparisons between pairs of groups were performed using the Student’s *t*-test. A *p*-value of less than 0.05 was considered statistically significant.

## Data Availability

The original contributions presented in this study are included in the article. Further inquiries can be directed to the corresponding author.

## Abbreviations

The following abbreviations are used in this manuscript:

Lf	Lactoferrin
bLf	Low-iron bovine lactoferrin
Holo-bLf	Iron-charged bovine lactoferrin
Caco2	Colonic cancer human cells
Gal/GalNAc	Galactose/N-acetyl galactosamine
TLR	Toll-like receptor
MDR1	Multidrug resistance 1
SN	Cell culture supernatant
CPs	Cysteine proteases
